# Natural product scores and fingerprints extracted from artificial neural networks

**DOI:** 10.1016/j.csbj.2021.07.032

**Published:** 2021-07-30

**Authors:** Janosch Menke, Joana Massa, Oliver Koch

**Affiliations:** a Institute of Pharmaceutical and Medicinal Chemistry, Westfälische Wilhelms-Universität Münster, Corrensstraße 48, 48149 Münster, Germany; b Center for Multiscale Theory and Computation, Westfälische Wilhelms-Universität Münster, Corrensstraße 48, 48149 Münster, Germany

**Keywords:** Natural products, Neural fingerprints, Similarity search, Virtual screening, Natural product likeness score, Neural networks, Autoencoder

## Abstract

Due to their desirable properties, natural products are an important ligand class for medicinal chemists. However, due to their structural distinctiveness, traditional cheminformatic approaches, like ligand-based virtual screening, often perform worse for natural products. Based on our recent work, we evaluated the ability of neural networks to generate fingerprints more appropriate for use with natural products. A manually curated dataset of natural products and synthetic decoys was used to train a multi-layer perceptron network and an autoencoder-like network. In-depth analysis showed that the extracted natural product-specific neural fingerprint outperforms traditional as well as natural product-specific fingerprints on three datasets. Further, we explored how the activations from the output layer of a network can work as a novel natural product likeness score. Overall, two natural product-specific datasets were generated, which are publicly available together with the code to create the fingerprints and the novel natural product likeness score.

## Graphical abstract

**Figure:**
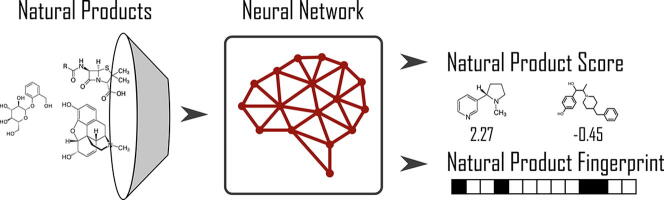

## 1 Introduction

Natural Products (NPs) constitute an important ligand class for medicinal chemists. Early usages of NPs date back to the ancients but even today they remain crucial as a source for novel drugs [1]. Between 1981 and 2010 alone, 34% of all small molecules approved by the FDA were either natural products or NP-derivatives. One reason for the success of NPs is that they are natural metabolites [2] and with this, they are expected to have an intrinsic permeability and bioavailability. This is a crucial property for most drugs and many interesting small molecules fail due to the lack thereof. Although natural products are a potent source of novel drug candidates, many cheminformatics methods were developed with synthetic compounds in mind, as they make up most of the compounds available in virtual libraries [3]. This can be an issue, as NPs are distinct from synthetic molecules and cover a different chemical space. They tend to have a higher scaffold diversity, are more rigid, included more fused ring systems, and have more chiral centers [4], [5], [6].

**Fig. 1:**
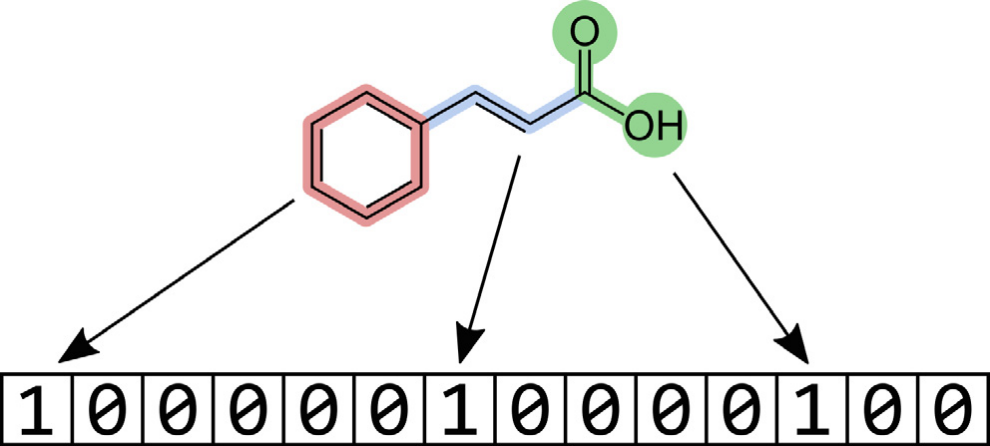
Example of a molecule being translated into a fingerprint. The presence of specific substructures is indicated by a “1” at a specific position on the vector.

A frequently employed strategy in the early stages of drug discovery is fingerprint-based virtual screening (FBVS). It builds on the assumption that similar compounds have similar properties [7]. FBVS aims to find novel potentially active compounds by screening through large chemical databases and identifying molecules that are similar to already known active compounds. The similarity between molecules is assessed by comparing molecular fingerprints. These fingerprints encode the molecular structure into a vector that is readable by the computer (see Fig. 1). Thus, by comparing fingerprints one can assess the similarity of the molecular structures. A wide variety of fingerprints exists, each with its unique advantages and drawbacks. The MACCS key 166 [8], for example, is a dictionary-based fingerprint that encodes predefined features into a vector of length 166. Features or substructures that are not included in the dictionary are not encoded in the fingerprint. Hashed fingerprints solve this issue by iteratively analyzing the substructures present in a molecule and using a hash function to generate a bit vector based on the identified substructures [9].

However, due to the above-mentioned characteristics of natural products many commonly used fingerprints are prone to struggle with the complexity of natural products [10] Seo and colleagues [11] addressed this issue by creating a natural product-specific fingerprint, which outperforms most regular fingerprints in the NP space. The fingerprint is built upon fragments frequently found in natural products. An alternative strategy could be derived from our recent work, using the activations of trained neural networks as a novel molecular fingerprint [12]. Given that a network is trained to predict the properties of molecules, one should expect that the activations of molecules with similar properties have similar activations in the deeper layers of the network. In our previous work, we showed that these neural networks can also be used to generate target-specific fingerprints implicitly incorporating target-relevant information [12]. A similar approach was chosen by Stojanović and colleagues [13] who trained a Graph Neural Network to predict the binding of molecules to a specific protein target. Later fingerprints were extracted from the network for a new dataset and it could be shown that these neural fingerprints outperformed traditional ones. Similar success was found by Fabian et al. [14] in which a transformer rather than a Graph Neural Network was used. Other groups have used the neural fingerprints as a tool to map reaction [15] or as an extra validation step in their prediction [16]. The strategy can also be adapted to encode molecules in a way that is more relevant to natural products and therefore lead to better results in virtual screening.

The aim of our work is to encode implicit natural product-relevant information while retaining enough information on the chemical space of such natural products. This can be achieved by training neural networks to distinguish natural products from synthetic ones. Additionally, the activations of the output layer are an estimate of how likely a given molecule is a natural product. This offer an alternative to existing measure such as the Natural Product Likeness (NPL) score [17], [18], [19]. A drawback to this approach is that it requires enough data to train and validate the neural network which is not always readily available. However, with the recent introduction of the Coconut database [20] this issue was solved for natural products. This database is publicly available and contains more than 400,000 natural products.

We used the Coconut database to create a new training dataset for natural product identification. Further, an FBVS validation set specifically for natural products is created from the NPASS library [21]. Based on these datasets, we show how simple feed-forward neural networks can be trained to produce molecular representations which are better suited for natural products and hence perform better than other molecular fingerprints when screening for such. In addition, a natural product score can also be extracted from the trained neural networks for the assessment of the natural product likeness of unknown molecules. The code are available at https://github.com/kochgroup/neural_npfp.

## 2 Methods

### 2.1 Data

#### 2.1.1 Training data

For the proper training of neural networks, a curated dataset consisting of natural products and synthetic molecules was generated. As a starting point for the dataset, we used the Coconut database [20] and calculated an updated version [18] of the natural product likeness (NPL) score originally introduced by Ertl et al. in 2008 [17]. Molecules for which the NPL score could not be calculated, duplicates, and molecules that could not be parsed by RDKit (Version 2020.09.1) [22] were removed. This led to a reduction of the number of compounds from an initial 426,916 to 419,441. As the goal is to train neural networks to distinguish between NPs from synthetic compounds, the dataset needed to include synthetic molecules. Using a similarity search for each of the natural products in the Coconut database, appropriate synthetic compounds were identified in the Zinc [23] “in-stock” library. This library was first prepared by removing stereo-information, duplicates, and compounds that could not be processed by RDKit or the NPL scorer. This process reduced the number of compounds from ten million to around seven million. For this reduced dataset, the NPL scores were calculated. The goal of the subsequent similarity search was to identify synthetic compounds which are similar to natural products but have a lower NPL score. We used the FPSim2 library [24] for the identification of the most similar compounds in the Zinc dataset. The similarity search was performed with the ECFP4 (2048 bits) [25] and the Tanimoto similarity. The most similar molecules were then filtered based on their NPL score and only Zinc molecules with an NPL score below zero were accepted. The reason for excluding compounds from the ZINC with high NPL scores was to avoid integrating compounds that are thought to be synthetic but are actually natural products or derivatives.

Additionally, the synthetic compounds were required to have a similarity of at least 0.5 to the query. This should ensure that the structural differences between the natural and synthetic compounds do not get too large, which could make the task too easy for the neural network. If the two mentioned conditions were met the identified compound was accepted into the dataset. This step was repeated for every single natural product in the Coconut database. The number of added molecules was limited to the ten most similar per natural compound. This should prevent natural products for which many similar compounds exist to dominate the decoy dataset. After completing the similarity search, duplicates were removed again and all identified synthetic compounds were screened against the Zinc database of biolites to ensure that no NPs are accidentally included in the synthetic dataset.

Lastly, the data was screened for molecules that are also included in one of the external validation sets. These molecules were removed from the training data. Further, we computed surface descriptors for all molecules in the training dataset using the package mordred [26]. The list of surface descriptors can be found in the Supplementary Information. For all molecules in the dataset, we calculated the ECFP4 with a size of 2048 using RDKit.

**Fig. 2:**
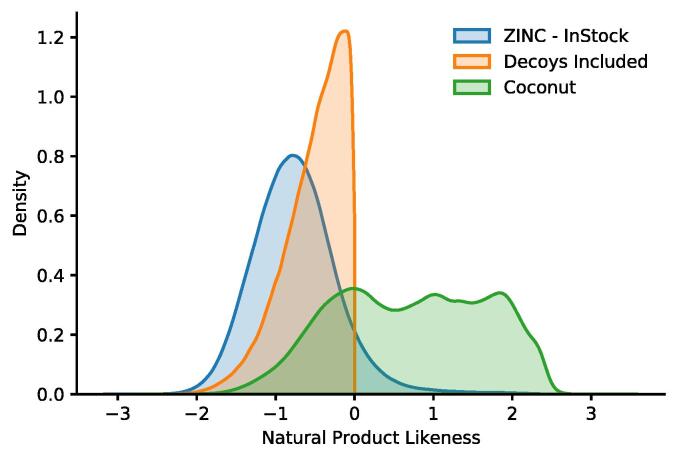
Distribution of Natural Product Likeness in the Coconut (n  = 394,939) and Zinc (n  = 7,158,026) and the decoys (n  = 210,412).

The final dataset consists of 394,939 natural products obtained from the Coconut database and 210,412 synthetic compounds from the Zinc database. In Fig. 2, a comparison of the different NPL scores is shown. While a overlap between the Zinc (in-store) and the Coconut database exists, large NPL scores can only be found for the Coconut database. Most lower NPL-score molecules belong to the Zinc database. Only a small fraction of the seven million original compounds made it into the final dataset. The reason for this is that the NPL score is based on substructures found in NPs. Due to this fact, it is difficult to find synthetic compounds similar to NPs but with a much lower NPL score. After the similarity search, we only retain molecules with an NPL of below zero which showed in the dataset itself.

#### 2.1.2 External Validation Data for Similarity Searches

For the external validation of the neural fingerprints, three different additional datasets were used. For this, data gathered by Seo et al. [11], which consists of two separate tasks, was retrieved. One dataset (in the original work named “Task 1”) is concerned with differentiating *between* synthetic and natural products. For more clarity, this task will be referred to as “NP Identification”. The second dataset (“Task 2”) consists of activity data on seven targets. Here only active and inactive natural products are included. The fingerprint is evaluated based on its ability to distinguish active from inactive natural products and assesses the fingerprint’s ability to distinguish *within* the class of natural products. This task will be referred to as “Target Identification”. These datasets required no further processing besides parsing the provided SMARTS via RDKit. In this process, one molecule had to be removed as RDKit was not able to convert the SMARTS into a valid SMILES. The third dataset was curated by us to create a situation that combines the “NP” and “Target Identification” challenges. This should evaluate the fingerprints on a more “realistic” dataset, consisting of active and inactive natural products as well as active and inactive synthetic compounds. The starting point was the selection of targets for which the NPASS library [21], another curated natural product library, had enough active as well as inactive natural products recorded. From Chembl [27], additional similar synthetic active and inactive compounds were added, using 300 nM as a cutoff value to decide which molecules are considered active or inactive. Unfortunately, the specific type of measure of potency was not always recorded, so 300 nM was the cutoff value for K_i_ and IC50 measures even though they are not measuring the identical concept. An overview of the three datasets and their tasks can be found in Table 1.

**Table 1:** Overview of the different datasets used for the validation of the fingerprints.

Task	Description	*n* Natural	*n* Synthetic	*n* Natural Active	*n* Synthetic Active	*n* Targets	Total n Compounds	
NP Identification*	Distinguish between natural and synthetic compounds	1000	1000	-	-	-	1000	
Target*	Distinguish between active and inactive natural products	896	0	312	0	7	896	
NP & Target	Identify active natural products among active and inactive synthetic compounds and natural products	2321	91002	444	1877	14	93323	

*published by Seo et al.*[*11*]**.*

### 2.2 Modeling

#### 2.2.1 Multi-layer perceptron

**Fig. 3:**
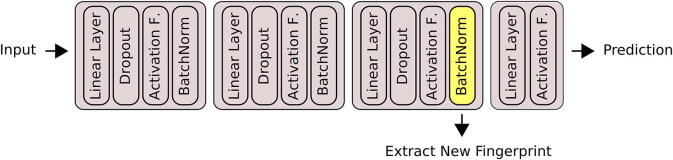
Schematic overview of the neural network architecture. From the last hidden layer, more specifically after the batch-normalization has been applied, the fingerprint is extracted. An additional linear transformation plus activation function are applied to the fingerprint before the network makes a prediction.

**Fig. 4:**
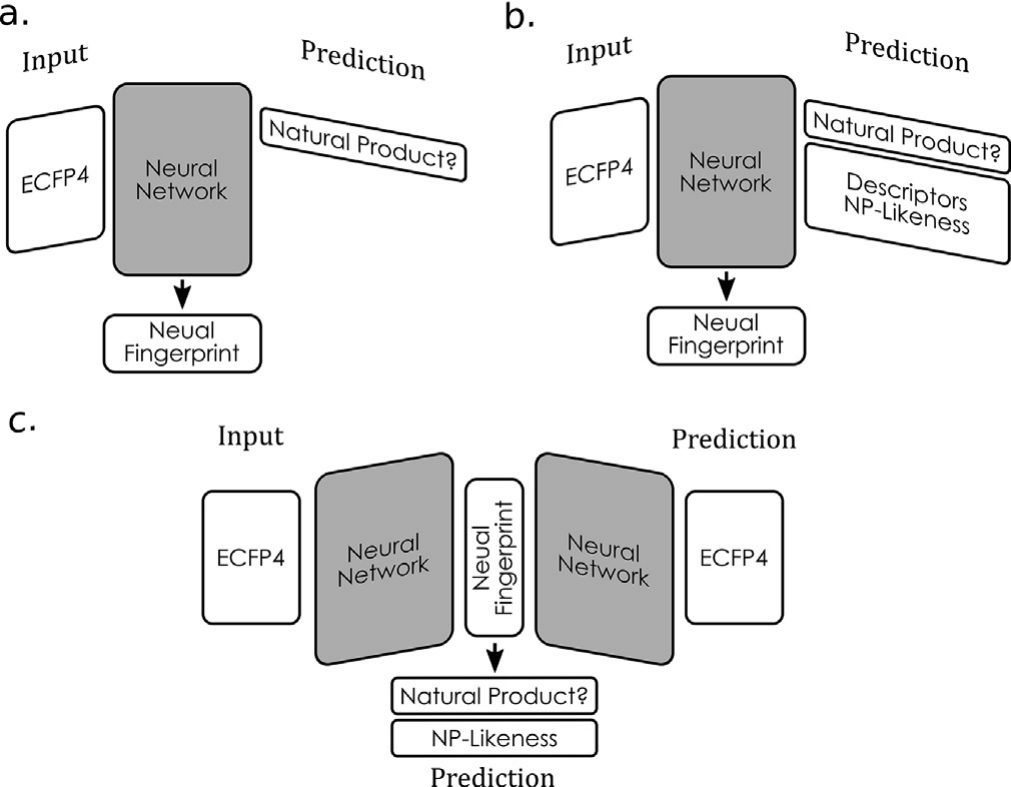
Comparison of neural network architecture. *a.* The Baseline model is trained to only predict whether a compound is a natural product. *b.* NP_AUX predicts additional surface descriptors. *c.* autoencoder-like NP_AE aims to encode the ECFP into a neural fingerprint from which the ECFP should be reconstructed. Additionally, the fingerprint is used to predict whether a compound is a natural product.

The core idea of the here proposed approach is that the activations obtained from a layer within a neural network can be used as a fingerprint. The deeper the layer is from which the activations are extracted the more relevant the fingerprint should be to the task that the network has been trained for. In our case the fingerprint is extracted from the last *hidden* layer in the network (see Fig. 3). Initially, a neural network was trained to solely predict whether a given compound is a natural product or not. This was chosen as a baseline model (see Fig. 4a). An extension of the baseline model (NP_AUX) is based on work by Fabian et al. [14], and predicts 48 additional surface descriptors next to whether a compound is a natural product or not (see Fig. 4b). The reason for predicting additional descriptors is that the baseline model might neglect too much structural information of the molecule. By including these extra predictions the network has to create more nuanced activations which can result in a more expressive fingerprint.

#### 2.2.2 Autoencoder

The third model (NP_AE) mimics an autoencoder, often used for language models, which aims to encode the ECFP into a dense lower-dimensional vector and then decode the original ECFP from this dense vector (Fig. 4c). On top of reconstructing the ECFP, the network is trained to distinguish NPs from synthetic compounds based on this dense vector in between the encoder and decoder. Thus, a loss function for the reconstruction and one for the identification of natural products is used for training the network.

#### 2.2.3 Additional models

Multiple additional variation to the models were made, with the goal to improve the models’ performance. Among those is an adaptation of the Graph Convolution Network (GCN) by Kipf et al. [28] As none of those lead to improvements of the here introduced models, the description and results can be found in the Supplementary Information.

#### 2.2.4 Model training

All models were build using PyTorch (Version: 1.7.0) [29]. For training, the ADAM algorithm in combination with the One Cycle Learning Rate policy is used. Each model was trained five times using a random split 5-fold cross-validation. Where each training set consisted of 80% of data and 20% was used for the validation set.

Following each linear layer, Dropout and Batch Normalization are applied (see Fig. 3). The Rectified Linear Unit (ReLU) activation function is used after each linear layer, only after the fingerprint layer, the hyperbolic tangent (Tanh) is applied instead. We chose the size of the fingerprint layer to be 64 and hence the size of the extracted fingerprint will also be 64. For a more detailed overview of the architecture and choice of hyperparameters, we refer to Table s6 in the Supplementary Information. We decided against extensive hyperparameter optimization as this study aims to prove the general idea of generating natural product encodings, rather than generating the best natural product neural fingerprint possible. We focus on using relatively simple neural networks to achieve improved results.

As the predictions include both classification and regression tasks, different loss functions are required. The Binary Cross-Entropy Loss is used for all classification tasks and the Root Mean Squared Error is used for all regression tasks. For the NP_AUX model, the two loss functions are weighted relative to the number of tasks, so that each individual task contributes equally to the overall loss. For training the NP_AUX we only required the BCE Loss. However, to prevent the task of identifying natural products from being overshadowed by the loss from reconstructing the ECFP, we switched the weight ratio from originally 1:2048 to 1:100. Thus the loss of identifying natural products weights 20 times more than reconstructing a single bit of the ECFP.

### 2.3 Validation

#### 2.3.1 Model Validation

The trained neural networks were evaluated based on the performance on the validation set obtained by splitting the training data into training and validation sets. As we opted for a 5-fold cross-validation the average across the five folds is reported together with the standard deviation. These results are only to show that from their predictive quality the three models (Baseline, NP_AUX, and NP_AE) perform similarly in identifying Natural Products.

#### 2.3.2 “NP Identification”

In the original article by Seo and colleagues, no AUC for their fingerprint was provided for the “NP Identification” task, but we estimated it using a trapezoid approximation based on the confusion matrix from the article. We aimed to reproduce their evaluation method by performing a k-nearest-neighbor (k-nn) classification with the fingerprint as input. More specifically compounds are classified based on the single compound they are most similar to. Thus, resulting in a 1-nn classification. Ten-fold cross-validation is used and the mean performance is reported. The cosine similarity was used as a measure of similarity for the neural fingerprint.

#### 2.3.3 “Target Identification”

The original analysis of the NC_MFP by Seo et al. [11] using the 1-nn was done via a paid software and we were unable to reproduce the original results from the article using Python for this particular task. As an alternative validation strategy for the “Target Identification” task, we chose to perform a similarity search in which every active molecule is picked as query ones and perform a similarity search. The performance was evaluated based on the AUC and Enrichment Factor (EF) 1%.

#### 2.3.4 “NP & Target Identification”

In the last step, the performance of the neural fingerprint on the “NP & Target” dataset was analyzed. For each of the 14 targets similarity searches are performed. Active molecules from the NPASS library were used as queries. The NC_MFP was not included in this analysis, as we were not able to produce fingerprints with the code provided by Seo et al. The performance on the similarity search was evaluated based on the AUC and ${\mathrm{EF}}_{1\%}$. The similarity was assessed using the Tanimoto similarity for the ECFP and the cosine similarity for the neural fingerprints.

#### 2.3.5 Metrics

**Area under the Curve** The Area under the Curve (AUC) measures the area underneath the receiver operating characteristics (ROC) curve and is used as a measure of a model’s performance in classification problems. The AUC-ROC ranges from zero to one. Where an AUC-ROC of 1 indicates a perfect classification by the model. A value of 0.5 indicates that the model performs equal to guessing randomly to which class a data point belongs. One can calculate the AUC-ROC by using the true-positive and false-positive rate shifting the decision threshold for the binary classification. At each threshold, the false-positive rate is plotted against the true-positive rate. For future reference, we will use the Term *AUC* to refer to the AUC-ROC. The AUC is used both for the evaluation of the predictive performance of the model, but also for evaluating the performance of the fingerprint in the similarity search. Here, the AUC measures how often active molecules are more similar to a query than inactive molecules. A perfect AUC would be obtained if every active molecule is more similar than all inactive ones.

**Enrichment Factor** In the similarity search, we also make use of a second measure called the Enrichment Factor (*EF*). Similar to the AUC it measures how well active molecules are identified by similarity, with the distinction that it focuses only on a certain percentage of similar molecules. In a real-life application, databases contain millions of compounds and most users will only look at the top fraction of most similar molecules. For that reason the performance of a fingerprint in the Top x% is important. The ${\mathrm{EF}}_{1\%}$, thus refers to the performance in the Top 1%. It measures how much more likely it is to find an active molecule in the Top 1% compared to picking a molecule randomly. It is computed as follows ${\mathrm{EF}}_{x\%}=\frac{N_{x\%}^{\mathrm{active}}}{N_{x\%}^{\mathrm{total}}}/\frac{N_{100\%}^{\mathrm{active}}}{N_{100\%}^{\mathrm{total}}}$.

### 2.4 Additional applications

#### 2.4.1 Natural product scores

The NPL score by Ertl et. al. [17] indicates how likely a molecule is a natural product. Similarly, the activation of the neuron that is responsible for predicting natural products in a neural network can also be used as a measure for the probability of being a natural product. Thus, these activations can be thought of as a sort of natural product likeness score. We compare how Ertls NPL score evaluates various structures to the score obtained by our neural networks.

#### 2.4.2 Pseudo natural products

As a last comparison, the models were applied to some newly proposed pseudo-natural products developed by Waldmann and his coworkers [30], [31]. These pseudo-NPs are designed to create NP-like structures outside of the traditional chemical space of natural products. This is achieved by combining fragments often found in NPs via a variety of different linking methods. 15 compounds from the original paper by Karageorgis et al. [30] were picked (in the original paper compounds 22–36). The fingerprints for the pseudo-NPs were evaluated by calculating and comparing the similarities between the 15 selected molecules from Karageorgis et al. [30] Subsequently, for each compound a similarity search was performed on the Zinc In-stock library using ones the ECFP and ones the neural fingerprint.

## 3 Results & discussion

### 3.1 Model performance

We trained three network architectures to distinguish between natural and synthetic compounds. How well the model distinguishes between those two classes is shown in Table 2. We also report the AUC specifically for all compounds with an NPL score below zero, as this space is populated by both synthetic as well as natural compounds. We can see that all models perform well at classifying natural products. The differences between the models are only marginal. The performance of all models also degrades similarly for low NPL molecules. Here, a drop of 0.06 points is observed for each of the models. Also, the standard deviation across the 5-folds of the data is small, indicating that training these models leads to stable results.

**Table 2:** Results of the models identifying natural products within the validation set of the training data. The mean and standard deviation of the AUC across the 5-folds are reported

Model	AUC (SD)	AUC NPL < 0 (SD)
NP_AUX	0.9692 (0.0005)	0.9051 (0.001)
NP_AE	0.9659 (0.0006)	0.8935 (0.0015)
Baseline	0.9667 (0.0006)	0.8994 (0.0015)

### 3.2 Natural product score

**Fig. 5:**
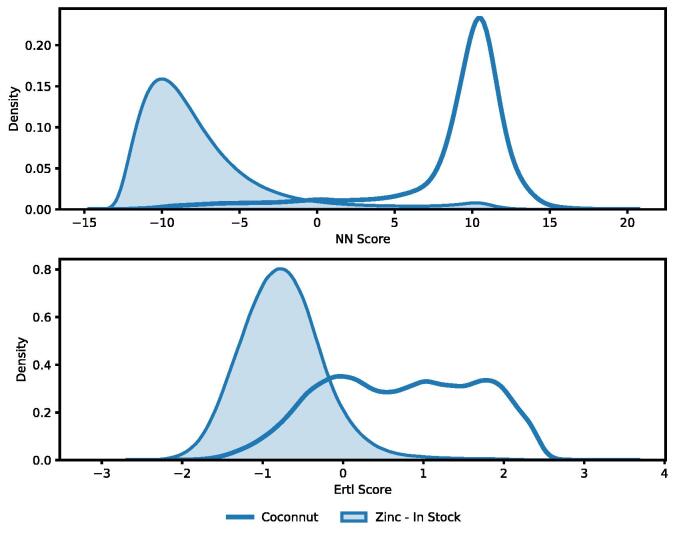
Comparing the distribution of NPL Scores between the proposed neural network based score (NN Score) and Ertl et al. on the Zinc “In-Stock” and Coconut database. For the Coconut dataset NN-scores are only shown for molecules that were not part of the training set.

In Fig. 5 the distribution of the here proposed neural natural product score is compared to the score introduced by Ertl et al. [17]. It becomes apparent that the neural network was trained on a binary classification task. The distribution of both datasets is much stronger separated for our natural product score than for Ertl‘s. Not only is the range of values much larger, but most compounds are also located at either end of the spectrum. This strong separation can be linked to the way the predictions of neural networks are made. A sigmoid function is applied to these scores, which scales them between zero and one. Large values are getting scaled close to one (being a natural product) and small values get scaled to zero (not a natural product). Only molecules for which the model is unsure about are receiving values in between. With a well-performing model, one should expect such distribution. For the Zinc dataset, both our and Ertl’s NPL distribution share similarities. They appear to be normally distributed with the score from the neural network being slightly skewed to the left. The variance of these two scores is also relatively similar. This does not hold for the Coconut dataset. The neural natural product score is distributed normally with a skew to the right, while the Ertl score is more uniformly distributed across a larger range for values.

**Fig. 6:**
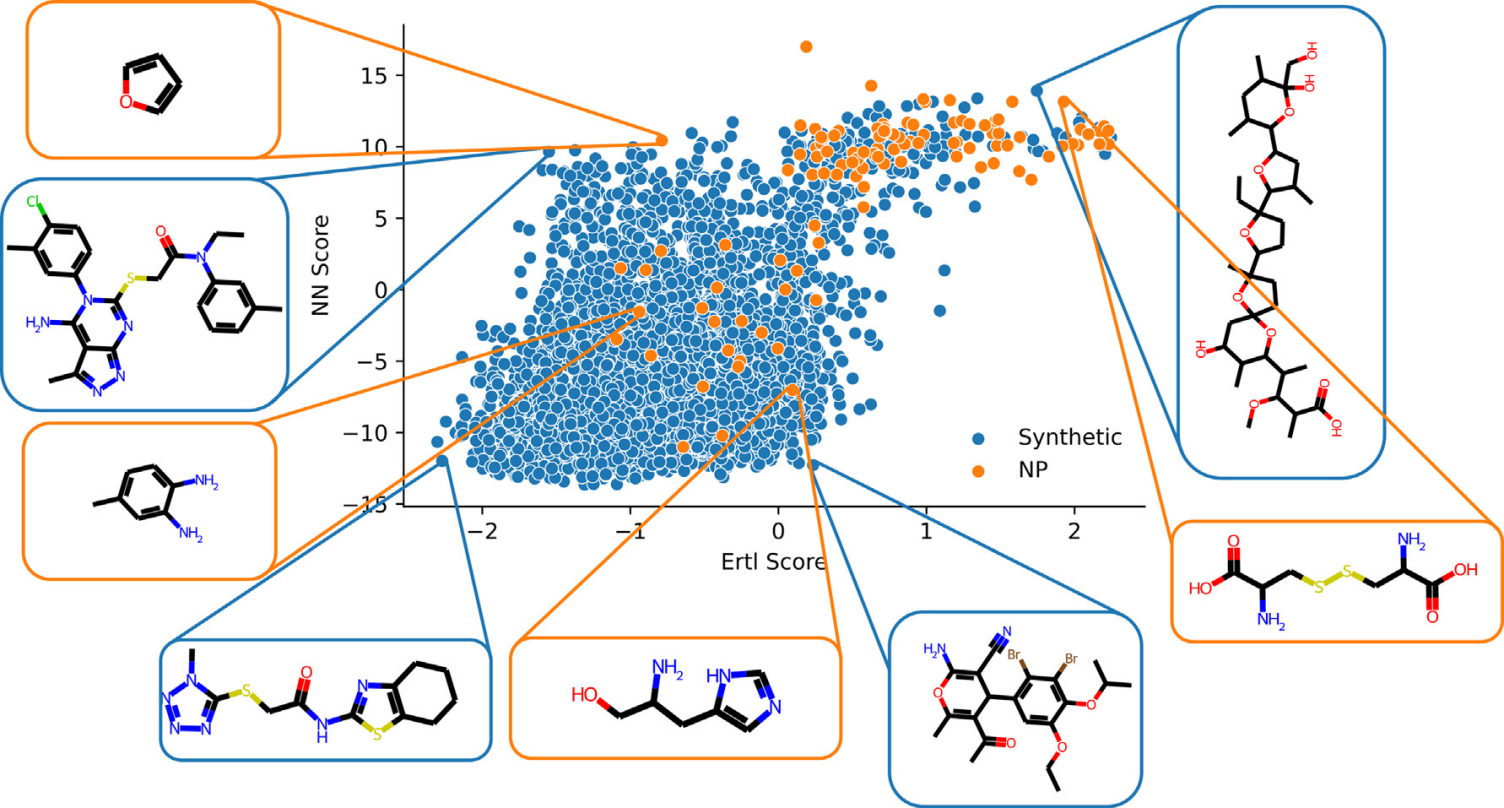
Natural product likeness scores for compounds included in the ROR-Gamma subset. A comparison between the NPL score by Ertl et al. and the here proposed neural network (NN) score is made.

For a more in-depth comparison the natural product likeness of Ertl was plotted against the natural product score obtained from the neural network (see Fig. 6). This was done on a subset of compounds from the validations set used for the “NP & Target” task. The majority of natural compounds are ranked high by both Ertl’s as well as the neural network score. Both scores also seem to agree for lower NP-like natural products. Molecules that have drastically different scores are highlighted in Fig. 6. Across the dataset, however, the agreement between the two scores is only moderate with a correlation of 0.4603. Further, the comparisons show that Ertl’s score varies more for high-scoring natural products than ours, which hints at the fact that Ertl’s score can evaluate natural products more nuanced.

Correlations between some basic molecular properties and natural product scores were calculated to explore what drives the difference in scores between molecules. The results can be found in Table 3. Molecular weight has some small correlation for the neural network score but the influence is close to zero for Ertl’s. This is not surprising, as Ertl’s score is based on the relative frequency of occurring fragments, which ensures that the size of molecules does not affect the score. The neural network does not take that into account, and hence the influence of molecular weight is larger. Second, the relation between the ratio of heteroatoms and NPL was evaluated. Here the effect is close to zero for our score, but for Ertl’s score, there is a small negative relationship. Lastly, the relationship between the relative occurrence of SP^3^ carbons is evaluated. For both scores, the effect is almost identical. A small positive correlation between the fraction of SP^3^ carbons and the natural product score. Thus, molecules with the higher relative occurrence of SP^3^ carbons tend to rank higher.

**Table 3:** Correlation between different molecular properties and the NPL scores.

Property	Ours	Ertl et. al.
Molecular Weight	0.23	−0.026
Ratio Heteroatoms	−0.073	−0.282
Ratio SP3 Carbon	0.238	0.291

Overall, there is a moderate agreement between Ertl’s “handcrafted” natural product score and the score that was obtained from the neural network. Most natural products rank high for both scores, and both rank the same natural products lower. A big difference is that for natural products the neural network scores do not vary as much as Ertl’s score. This might be an issue as it limits its ability to distinguish between natural products with high NP likeness. A second difference is a smaller overlap between synthetic and natural products for the neural network score, which can be attributed to the binary task the network was trained for. In general, there is a small but important difference in definition between Ertl’s natural product *likeness*, and our natural product score. For Ertl, the goal was to create a score that evaluates the NP likeness, while the network was trained to identify natural products. Thus, the neural network score evaluates something slightly different, it evaluates how likely a molecule is a natural product rather than how alike it is to a natural product. A good example of this difference is Furan which is ranked high by the network score but low by Ertl’s score. It is defined as a natural product but does not have many specific NP-like features. A big advantage of the neural network score is that it does not assume that substructures have a linear additive effect on the natural product score. Ertl’s score is calculated by summing up the natural product likeness for all found fragments. This might be an issue for some combination of fragments that do not have a linear additive relationship between each other. Two fragments could often be found in natural products but just in the same molecule. This could be picked up by the neural network score but not by Ertl’s. However, Ertl’s score also has the advantage, that the scores transition appears to happen more linear, which might be easier to better suited for other deep learning methods such as generative deep learning models which optimizing molecules with regards to specific properties [32], [33]. In the end, there appears to be no clear winner between the two scores, while there is some overlap both scores have their unique advantage and further investigation is needed to elucidate in which situation which scores should be used.

### 3.3 Fingerprints and similarity search

#### 3.3.1 “NP Identification” dataset

The results for the NP Identification task are shown in Table 4. Generally, all three neural networks outperform the NC_MFP at distinguishing natural compounds from synthetics ones. Using the neural network directly to make the prediction is superior to the use of the extracted fingerprints in combination with a nearest-neighbor search. A reason for this is that the neural network has an additional layer to extract information from the fingerprint, which allows it to weight the input more nuanced. Second, in the paper, a k-nn with k=1 is used, which runs the risk of overfitting. This could be avoided with larger values for *k*. The Model AUC, the classification made by the network, is again almost equal for all three models. The best fingerprints are obtained by NP_AE Model, it outperforms the baseline by 3.2% and AUX model by 0.6%. The NP_AUX still provides on average 2.5% better AUC compared to fingerprints of the baseline model. This indicates that the inclusion of the additional tasks, like predicting descriptors or the ECFP, improves the fingerprint.

**Table 4:** Comparison of AUC in distinguishing between synthetic and natural compounds in the “NP Identification” dataset.

Fingerprint	Model AUC	Fingerprint AUC
NC_MFP	-	0.747
NP_AUX	0.947 (0.002)	0.874 (0.005)*
NP_AE	0.942 (0.001)	0.88 (0.002)*†
Baseline	0.944 (0.001)	0.852 (0.006)

Significant difference to the: *Baseline;†*NP*_*AUX. (*α=0.05*).*

#### 3.3.2 “Target Identification” dataset

**Fig. 7:**
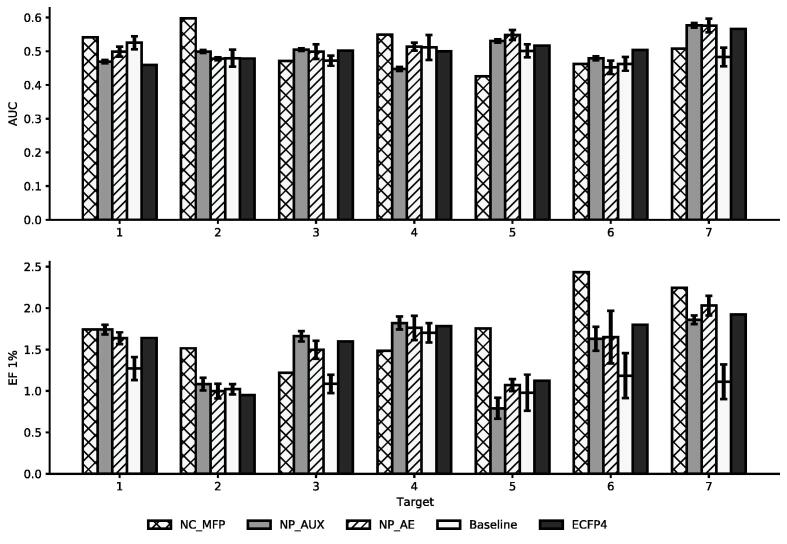
AUC and Enrichment of different fingerprints “Target Identification” dataset. For each target multiple similarities searches are performed and the average performance is displayed. The error bars indicate the standard deviation across the 5-folds.

Next, we looked at the performance of the fingerprints on the “Target Identification” task. This task involves identifying active NPs on seven different targets. In Fig. 7 the AUC and ${\mathrm{EF}}_{1\%}$ for each target and fingerprint are shown. Considering that an AUC of 0.5 indicates a performance that is equal to making a random choice, all fingerprints do not perform well at differentiating between actives and inactives. For the ${\mathrm{EF}}_{1}\%$ the NP_AUX, NP_AE, and ECFP4 only have small differences between each other and behave similarly across the seven targets. This indicates that these neural fingerprints are still related to the ECFP4, even though they got transformed by passing through a neural network. While the baseline fingerprint performs on par with the other fingerprints on some targets on others it performs noticeably worse.

This effect is more pronounced when looking at the average enrichment in Table 5. Across the seven targets, its enrichment is significantly lower than that of any of the other fingerprints. The NC_MFP provides on average the highest enrichment, but the difference to the other fingerprints is not significant. Overall it becomes apparent that all fingerprints perform similarly well on this task. Only the baseline model performs worse than all other fingerprints. For almost all targets it provides the worst enrichment.

**Table 5:** Average performance across the seven targets of fingerprints in the similarity search on the “Target Identification” task. The Standard Deviation refers to the average deviation across the 7 targets.

	AUC (SD)	EF 1% (SD)
NC_MFP	0.508 (0.055)	1.77 (0.399)*
NP_AUX	0.501 (0.04)	1.512 (0.379)*
NP_AE	0.509 (0.039)	1.521 (0.343)*
Baseline	0.491 (0.021)	1.194 (0.226)
ECFP4	0.504 (0.031)	1.545 (0.339)*

Significant difference to the: *Baseline. (α=0.05*).*

#### 3.3.3 “NP & Target Identification” dataset

**Fig. 8:**
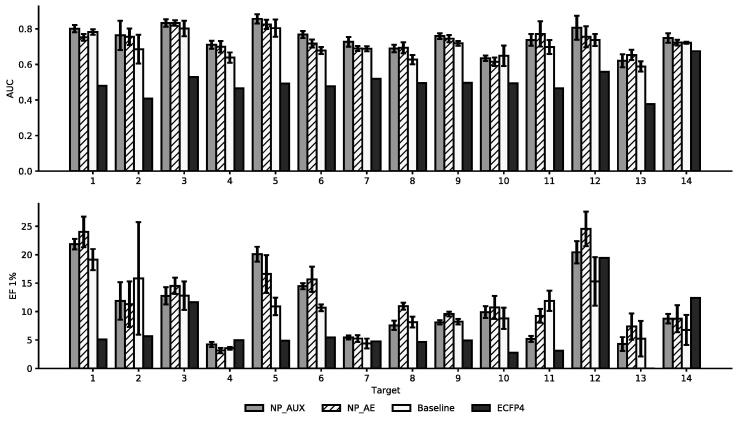
AUC and Enrichment of different fingerprints “NP & Target Identification” dataset. For each target multiple similarities searches are performed and the average performance is displayed. The error bars show the standard deviation across the 5-folds.

In Fig. 8 the results of the similarity search on our NP & Target Identification set are shown. Initial experiments showed that great performance on this dataset can already be achieved by simply ranking molecules by their NPL score, rather than their similarity to the query compound. As this is not the intended purpose, all natural products with an NPL score greater than one were removed from that dataset for the similarity search. The AUC for all neural fingerprints is almost 1.5 times as large as that of the ECFP4. This holds for almost all targets. Amongst the neural network approaches, the fingerprints of the NP_AUX and NP_AE provide the best AUC across the 14 targets. For the ${\mathrm{EF}}_{1\%}$ again the neural fingerprints outperform the ECFP4, only for a few targets the performance equal or slightly better.

Looking at the average performance across the 14 targets in Table 6, it becomes clear that the neural fingerprints are indeed superior in their ability to identify active natural products. The best fingerprints are obtained from the AE and AUX model. They find more active natural products both across the whole dataset as well as in the Top 1%. While the baseline model is better than the ECFP4 it lacks behind the other two neural network fingerprints.

**Table 6:** Average performance in the similarity search across all targets on the “NP & Target Identification” Task. The Standard Deviation refers to the average deviation across the 14 targets.

Model	AUC (SD)	${\mathrm{EF}}_{1\%}$ (SD)
NP_AUX	0.747 (0.066)•*	11.067 (5.902)•
NP_AE	0.731 (0.058)•*	12.265 (6.063)•*
Baseline	0.701 (0.063)•	10.115 (4.416)•
ECFP4	0.495 (0.066)	6.408 (4.735)

Significant difference to the: *Baseline; • *ECFP (*α=0.05*).*

**Fig. 9:**
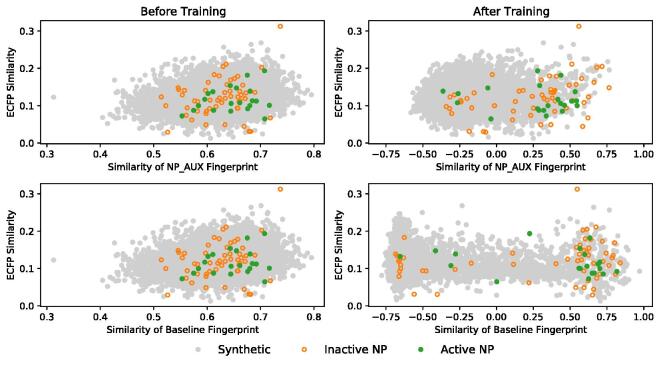
Similarity of the NP_AUX and Baseline versus the ECFP4 before and after training. Through the training process a separation between natural products and synthetic ones can be seen.

To further investigate how the training process changes the encoding of the molecules into a neural fingerprint we compared the ECFP4 similarities of compounds to the similarities obtained from the NP_AUX model and baseline model. A random target and active query were chosen and both the ECFP4 and neural fingerprint similarity were calculated for all other compounds in that dataset. The relationship between those similarities was compared with regards to changes before and after. The results are shown in Fig. 9. For both the baseline and the NP_AUX, the training leads to a separation of natural products and synthetic compounds. Meaning that after training, natural products tend to be more similar to a natural product query than before training. However, while for the NP_AUX fingerprint the separation is more gradual and natural products can be found at all levels of similarities, for the baseline fingerprint the separation is more dichotomous. Most natural products occupy the space of most similar molecules while only a few are found in the lower similarities. Interestingly something similar happens for the synthetic compounds, where the majority of compounds are either being very similar or very dissimilar. This resembles somewhat the binary classification (NP vs. synthetic) that the baseline model was trained for. The findings confirm the idea that the baseline fingerprint is not so nuanced in his representation of molecules, which in return explains the poor performance.

For completeness of the analysis, we also analysed if a Graph Convolution Network (GCN) [28] is able to generate better fingerprints than the here discussed models. However, on all task the GCN performed worse than the other architectures. This falls in line with earlier research of ours [12], and is not uncommon to occur. For example, Jiang and colleagues [34] showed that the Attention FP [35], a graph neural network providing state of the art results on many benchmark sets, performs only on par with descriptor-based models. The results of the GCN can be found in the Supplementary Information.

Overall, our results show that it is possible to use neural networks to generate more focused fingerprints from existing fingerprints. The ECFP4 is outperformed on almost every task by the neural fingerprints as well as the NC_MFP, making it the least suited fingerprint for the screening for natural products. Amongst natural product-specific fingerprints, the NC_MFP performs the worst, besides for the enrichment on the “Target Identification” task. This proves the validity of integrating natural product information into a fingerprint through the training of neural networks. The results show that predicting additional descriptors during training adds more “structural awareness” to the neural fingerprint, making them outperform the baseline neural fingerprint. While this lack of structural information did not matter when differentiating between synthetic and natural compounds, it appears to be important for tasks where distinctions have to be made within the chemical space of natural products. This is especially not-worthy as all three networks perform equally well at predicting natural products. Between the other two models not clear difference can be made out. The NP_AUX and NP_AE fingerprints perform similarly well with none of the two models clearly outperforming the other.

### 3.4 Pseudo natural products

**Fig. 10:**
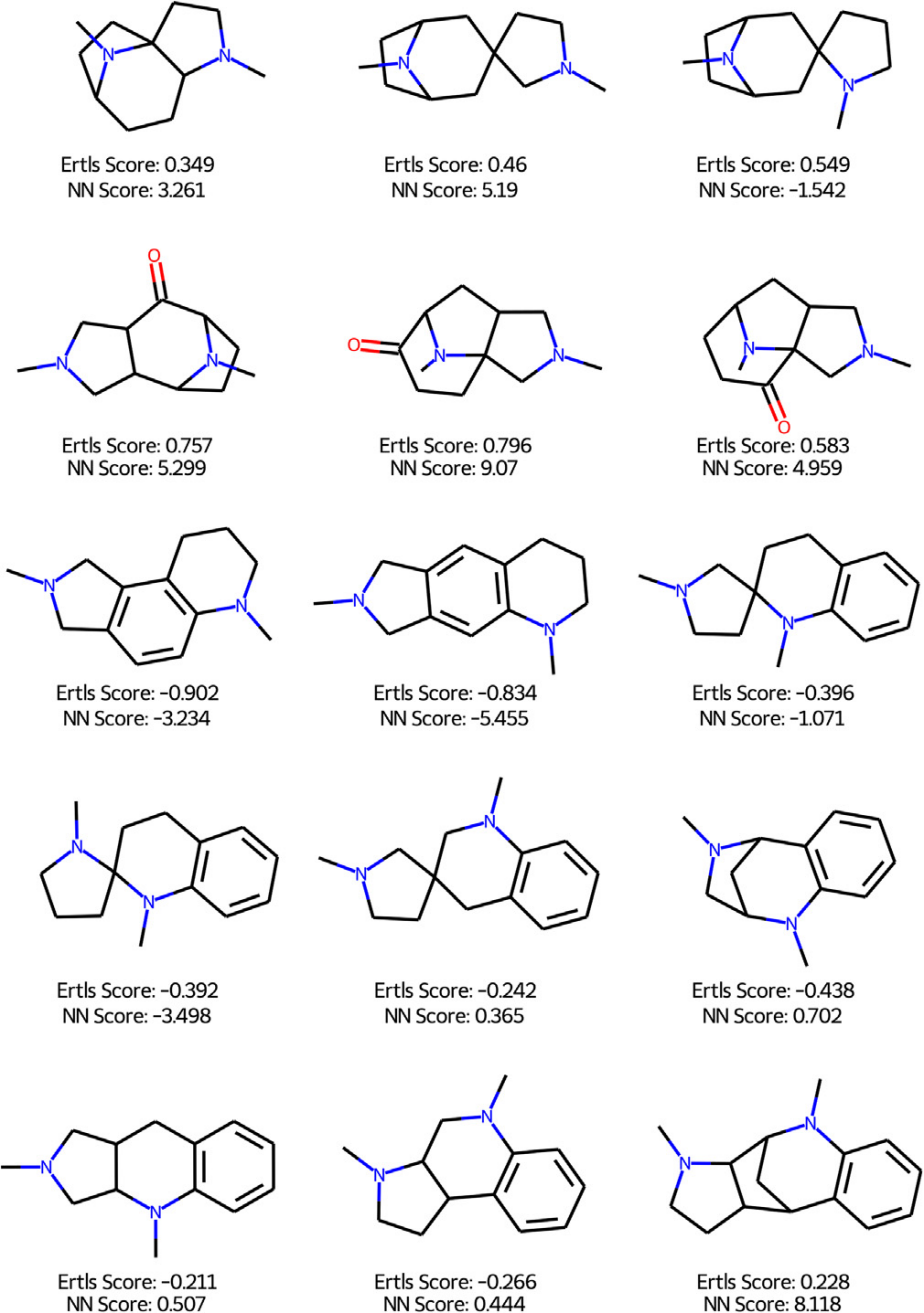
Comparison of Ertl‘s and the neural network natural product score on 14 Pseudo-Natural Products.

In Fig. 10 the 15 pseudo-NPs proposed by Karageorgis et al. [30] are shown with their respective Ertl NPL and our neural network score. Again the different distribution of values can be seen. Ertl‘s scores are closer together and the transition from scores is much smoother. In contrast, a small change can have a drastic impact on the here proposed neural network score. This again can be attributed to the binary training process, where only a few compounds occupy the space between natural and synthetic compounds. Molecules with a higher Ertl score tend to have a higher neural network score as well. The correlation between the two scores for these fourteen compounds is 0.804, much higher than the correlation on the ROR-Gamma dataset. This indicates that the neural network score performs similarly to Ertl’s score, even though these pseudo-natural products should lie outside of the neural product chemical space.

**Fig. 11:**
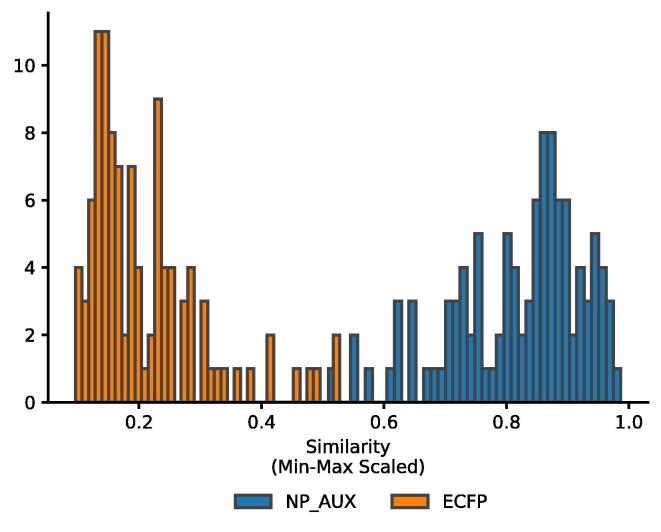
Similarity of the ECFP and NP_AUX between the 15 Pseudo-Natural Products.

When comparing the similarity amongst the Pseudo-NPs (See Fig. 11 it becomes apparent that the neural fingerprint estimates the similarity between the molecules higher than the ECFP. No single similarity for the ECFP exceeds 0.5, which means the ECFP considers the compounds dissimilar. The NP_AUX fingerprint always exceeds 0.5. This shows that the neural fingerprint evaluates natural products differently and can identify more similar natural products more easily.

**Fig. 12:**
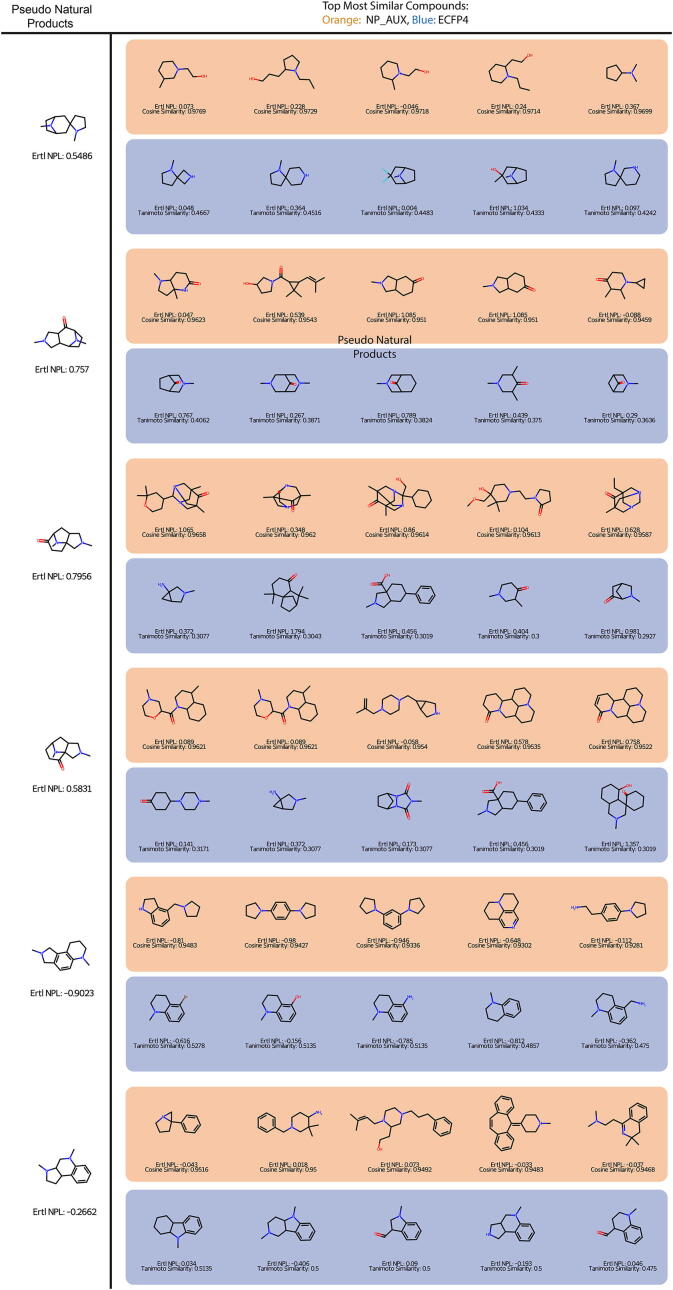
Compounds found through a similarity search. Top five most similar compounds found for pseudo-natural products in the Zinc dataset.

Lastly, a similarity search for the 15 pseudo-NP was performed once using the NP_AUX fingerprint and the ECFP4. This should pose an extra challenge to the neural fingerprints, as they were only trained on existing natural compounds, and pseudo-natural products were designed to leave that space. The results obtained by screening through the ZINC - In-Stock database are shown for a subset of six compounds in Fig. 12. The results for all pseudo-NPs can be found in the Supplementary Information.

Two things become immediately apparent. For one, the similarity for the ECFP is rather low while for the NP_AUX the similarities are quite high. In some cases, both fingerprints identify the same hit but the similarity is evaluated completely differently. Second, the NPL score tends to be higher for the compounds identified by the neural fingerprint in comparison to the ECFP4. Looking more specifically at the structures identified, the results are more mixed. For some compounds, the ECFP provides more sensible structures but there are other compounds for which the neural fingerprint provides a more diverse set, for example, including structures with additional rings. There is also some overlap found between the compounds, which can be attributed to the ECFP on which basis the neural fingerprint is trained. These results show, that both fingerprints analyze different chemical spaces but no clear difference in quality can be made out. However, the NP_AUX identifies molecules that are not identified by the ECFP4, thus different molecules are found by the neural fingerprint. Further, the general natural product likeness is higher with the neural fingerprint than with the ECFP4. Depending on the goal of the researcher this can be an important advantage. Overall, the fact that the molecules differ is an important point, as they could offer new starting points for researchers. Nevertheless the ECFP is also capable of providing reasonable suggestions, and it would be worth it to use a combination of these two fingerprints.

## 4 Conclusion

In this work, we set out to investigate the validity of generating fingerprints suited for the screening of natural products by training neural networks. In the process, we generated two datasets one for training the neural network and the second one for the validation of the fingerprint. It could be shown that it is indeed possible to use neural networks to generate fingerprints that are better than traditional fingerprints such as the ECFP or expert-crafted fingerprints such as the NC_MFP. The neural fingerprints rank natural products higher during similarity searches than other fingerprints. Our analysis showed that during training additional structure relevant endpoints should be included, as they allow the neural fingerprints to distinguish within the class of natural compounds. The best performing fingerprint was obtained from a network that was trained to identify natural products as well as additional surface descriptors. Further, we showed, that a natural product likeness can be extracted from the trained neural networks which constitute reasonable NP likeness scores. Thus neural network score was compared to the well-known NPL score developed by Ertl and colleagues. They both share similar properties and tend to evaluate compounds similarly. Due to the different calculations of their scores, they have unique properties which drive diverging evaluations for specific compounds. A direction for further improvement could be the pre-training of the neural networks on completely unrelated data. Here the focus is more on correctly predicting the descriptors or the ECFP and only in the final stage the models are trained to identify natural products. This could increase the structural awareness of the fingerprints. Both datasets and the trained models for similarity search and natural product scoring are available at https://github.com/kochgroup/neural_npfp.

## CRediT authorship contribution statement

**Janosch Menke:** Conceptualization, Data curation, Formal analysis, Investigation, Software, Validation, Visualization, Writing - original draft, Writing - review & editing. **Joana Massa:** Data curation, Investigation, Software, Writing - review & editing. **Oliver Koch:** Conceptualization, Formal analysis, Funding acquisition, Methodology, Project administration, Resources, Supervision, Validation, Writing - review & editing.

## Declaration of Competing Interest

The authors declare that they have no known competing financial interests or personal relationships that could have appeared to influence the work reported in this paper.
