# Supplementary material for: Natural product scores and fingerprints extracted from artificial neural networks
Source: Comput Struct Biotechnol J. 2021 Jul 30;19:4593–602. doi: 10.1016/j.csbj.2021.07.032 (PMC8445839; doi:10.1016/j.csbj.2021.07.032)
Supplement: Supplementary file 1 [file mmc1.pdf]

## Supplementary Information

### S1.1 Additional Models

Multiple additional adaptations to the models were made, with the goal to improve the models' performance. None of the adaptations lead to improvements. The adaptions are:

- **L1 - regularization**: The L1-Norm of the activations of the fingerprint layer was included as an additional part to the loss function. We scaled the Norm to a $10^{th}$ of its original value before adding it to the loss.

- **NPL>0** We trained the original models with a new dataset. This dataset also included compounds from the Zinc which had NPL scores above zero. We followed the same steps to remove duplicates and molecules which are known as natural products and or are included in any of the other validation sets. This lead to an increase of synthetic compounds from an initial 210,412 to 274,001 in the training set.

- **GCN** At last we investigated, how well a Graph Neural Network would be able to generate neural fingerprints. We chose an adaptation of the Graph Convolution Network (GCN) by Kipf and colleagues [28], which includes a more expressive read-out layer. The fingerprint layer is kept a size of 64 and is also the last hidden layer in the network.[12] The network is trained like the NP_AUX model. Thus it predicts the additional descriptors and whether a given compound is a natural product or not.

For all models, we evaluated the model performance and compared the fingerprints on the three validation tasks. For easier reading, the results of the original models are also included in the tables.

#### S1.1.1 Model Performance

**Table S1** Results of all models and their adaptation used for identifying natural products within the validation set of the training data. The mean and standard deviation of the AUC across the 5-folds are reported

| Model | AUC (SD) | AUC NPL < 0 (SD) |
|---|---|---|
| NP_AUX | 0.9692 (0.0005) | 0.9051 (0.001) |
| NP_AE | 0.9659 (0.0006) | 0.8935 (0.0015) |
| Baseline | 0.9667 (0.0006) | 0.8994 (0.0015) |
| NP_AUX + L1 | 0.9634 (0.0007) | 0.8948 (0.0011) |
| NP_AE + L1 | 0.9592 (0.0022) | 0.8675 (0.0068) |
| Baseline + L1 | 0.9654 (0.0007) | 0.8965 (0.0014) |
| NP_AUX + NPL>0 | 0.9333 (0.0005) | 0.8953 (0.0015) |
| NP_AE + NPL>0 | 0.9276 (0.0009) | 0.8873 (0.002) |
| Baseline + NPL>0 | 0.9311 (0.0008) | 0.8925 (0.0014) |
| GCN | 0.986 (0.0002) | 0.953 (0.0006) |

The use of the L1-Norm does not change much of the predictive power of the neural network itself. Only the NP_AE appears to perform a little worse for compounds with an NPL score below zero. The inclusion of synthetic compounds with higher NPL scores worsens the overall AUC. This is expected as the tasks became more difficult. The GCN performed noticeably better than any of the other models in identifying natural products.

#### S1.1.2 "NP Identification"

For the NP Identification, none of the adaptations lead to a strong change in results. Only the GCN works noticeably worse. Both the model and the fingerprint are beaten by any other architecture. This weak performance could be explained by overfitting to the training data which could also explain the exceptional model performance from before.

**Table S2** Comparison of AUC in distinguishing between synthetic and natural compounds in the "NP Identification" Task for the additional models and their adaptation.

| Fingerprint | Model AUC | Fingerprint AUC |
|---|---|---|
| NC_MFP | - | 0.747 |
| NP_AUX | 0.947 (0.002) | 0.874 (0.005) |
| NP_AE | 0.942 (0.001) | 0.88 (0.002 |
| Baseline | 0.944 (0.001) | 0.852 (0.006) |
| NP_AUX + L1 | 0.944 (0.003) | 0.864 (0.006) |
| NP_AE + L1 | 0.932 (0.002) | 0.792 (0.017) |
| Baseline + L1 | 0.939 (0.002) | 0.828 (0.006) |
| NP_AUX + NPL>0 | 0.941 (0.003) | 0.877 (0.006) |
| NP_AE + NPL>0 | 0.934 (0.004) | 0.878 (0.006) |
| Baseline + NPL>0 | 0.938 (0.002) | 0.843 (0.009) |
| GCN | 0.733 (0.051) | 0.735 (0.007) |

**Table S3** Average of all additional models performances across the seven targets of fingerprints in the similarity search on the "Target Identification" task. The Standard Deviation refers to the average deviation across the 7 targets.

| | AUC (SD) | EF 1% (SD) |
|---|---|---|
| NP_AUX | 0.501 (0.04) | 1.512 (0.379) |
| NP_AE | 0.509 (0.039) | 1.521 (0.343) |
| Baseline | 0.491 (0.021) | 1.194 (0.226) |
| NP_AUX + L1 | 0.503 (0.044) | 1.42 (0.362) |
| NP_AE + L1 | 0.475 (0.036) | 1.199 (0.273) |
| Baseline + L1 | 0.489 (0.023) | 1.135 (0.218) |
| NP_AUX + NPL>0 | 0.504 (0.037) | 1.458 (0.359) |
| NP_AE + NPL>0 | 0.514 (0.034) | 1.442 (0.293) |
| Baseline + NPL>0 | 0.499 (0.014) | 1.097 (0.209) |
| GCN | 0.504 (0.005) | 1.41 (0.026) |

**Table S4** Average performance for all models and their adaptations in the similarity search across all targets on the "NP & Target Identification" Task. The Standard Deviation refers to the average deviation across the 14 targets.

| Model | AUC (SD) | $EF_{1\%}$ (SD) |
|---|---|---|
| NP_AUX | 0.747 (0.066) | 11.067 (5.902) |
| NP_AE | 0.731 (0.058) | 12.265 (6.063) |
| Baseline | 0.701 (0.063) | 10.115 (4.416) |
| NP_AUX + L1 | 0.71 (0.063) | 9.643 (5.474) |
| NP_AE + L1 | 0.615 (0.054) | 7.97 (2.836) |
| Baseline + L1 | 0.705 (0.062) | 7.789 (4.029) |
| NP_AUX + NPL>0 | 0.644 (0.073) | 9.334 (5.406) |
| NP_AE + NPL>0 | 0.594 (0.058) | 10.175 (5.962) |
| Baseline + NPL>0 | 0.573 (0.072) | 9.425 (6.798) |
| GCN | 0.537 (0.072) | 5.047 (3.968) |

### S1.1.3 Target Identification

For the "Target Identification" task, all adaptions lead to a worse performing fingerprint, especially the L1-Norm leads to great reduction for the AE model. This time the GCN can beat the Baseline o both average AUC and $EF_{1\%}$

### S1.1.4 "NP & Target Identification"

In the "NP & Target" a similar story is repeated all adaptation leads to worse performance. The GCN performs much worse than any of the other models.

## S1.2 Targets

## Descriptors

EStat_VSA1, EState_VSA10, EState_VSA11, EState_VSA2, EState_VSA3, EState_VSA4, EState_VSA5, EState_VSA6, EState_VSA7, EState_VSA8, EState_VSA9, LabuteASA, PEOE_VSA1, PEOE_VSA10, PEOE_VSA11, PEOE_VSA12, PEOE_VSA13, PEOE_VSA14,

**Table S5** Number of active and inactive compounds in the "NP & Target Identification" dataset split by target

| | Target | NP Active | Synthetic Active | NP Inactive | Synthetic Inactive | Total |
|---|---|---|---|---|---|---|
| 1 | ROR-Gamma | 30 | 2008 | 81 | 5742 | 7861 |
| 2 | MAPT | 13 | 1198 | 43 | 6188 | 7442 |
| 3 | Acetylcholesterase | 31 | 1759 | 279 | 3916 | 5985 |
| 4 | PPARD | 20 | 282 | 46 | 267 | 615 |
| 5 | H3-K9-HMTase 6 | 39 | 1520 | 103 | 6770 | 8432 |
| 6 | Prelamin-A/C | 72 | 2426 | 47 | 5157 | 7702 |
| 7 | Androgen Receptor | 90 | 976 | 200 | 1569 | 2835 |
| 8 | DNA AP lyase | 68 | 3278 | 141 | 6192 | 9679 |
| 9 | Geminin | 199 | 3130 | 582 | 6965 | 10876 |
| 10 | ALDH1A1 | 11 | 785 | 312 | 7516 | 8624 |
| 11 | NRF2 | 63 | 1679 | 470 | 6282 | 8494 |
| 12 | VDR | 14 | 175 | 37 | 4512 | 4738 |
| 13 | TAD5 | 21 | 1459 | 27 | 7380 | 8887 |
| 14 | CYP 3A4 | 16 | 627 | 200 | 4825 | 5668 |

PEOE_VSA2, PEOE_VSA3, PEOE_VSA4, PEOE_VSA5, PEOE_VSA6, PEOE_VSA7, PEOE_VSA8, PEOE_VSA9, SMR_VSA1, SMR_VSA10, SMR_VSA2, SMR_VSA3, SMR_VSA4, SMR_VSA5, SMR_VSA6, SMR_VSA7, SMR_VSA8, SMR_VSA9, SlogP_VSA1, SlogP_VSA10, SlogP_VSA11, SlogP_VSA12, SlogP_VSA2, SlogP_VSA3, SlogP_VSA4, SlogP_VSA5, SlogP_VSA6, SlogP_VSA7, SlogP_VSA8, SlogP_VSA9, TPSA

## Hyperparameters

**Table S6** Hyperparameters used for training of the models. **Bold** indicates the fingerprint layer.

| Hyperparameters | Baseline | NP_AUX | NP_AE |
|---|---|---|---|
| Layer Size | [2048,1024,1024,**64**,1] | [2048,1000,1000,**64**,50] | [2048,512,**64**,512,2048][**64**,2]* |
| Dropout | 0.2 | 0.2 | 0.2 |
| Epochs | 20 | 20 | 300 |
| max LR | 0.0005 | 0.0005 | 0.0001 |
| Batchsize | 128 | 128 | 128 |
| Early Stopping | Yes | Yes | Yes |

**additional layer that is used to make predictions based on the neural fingerprint*

## Similarity Search Pseudo Natural Products

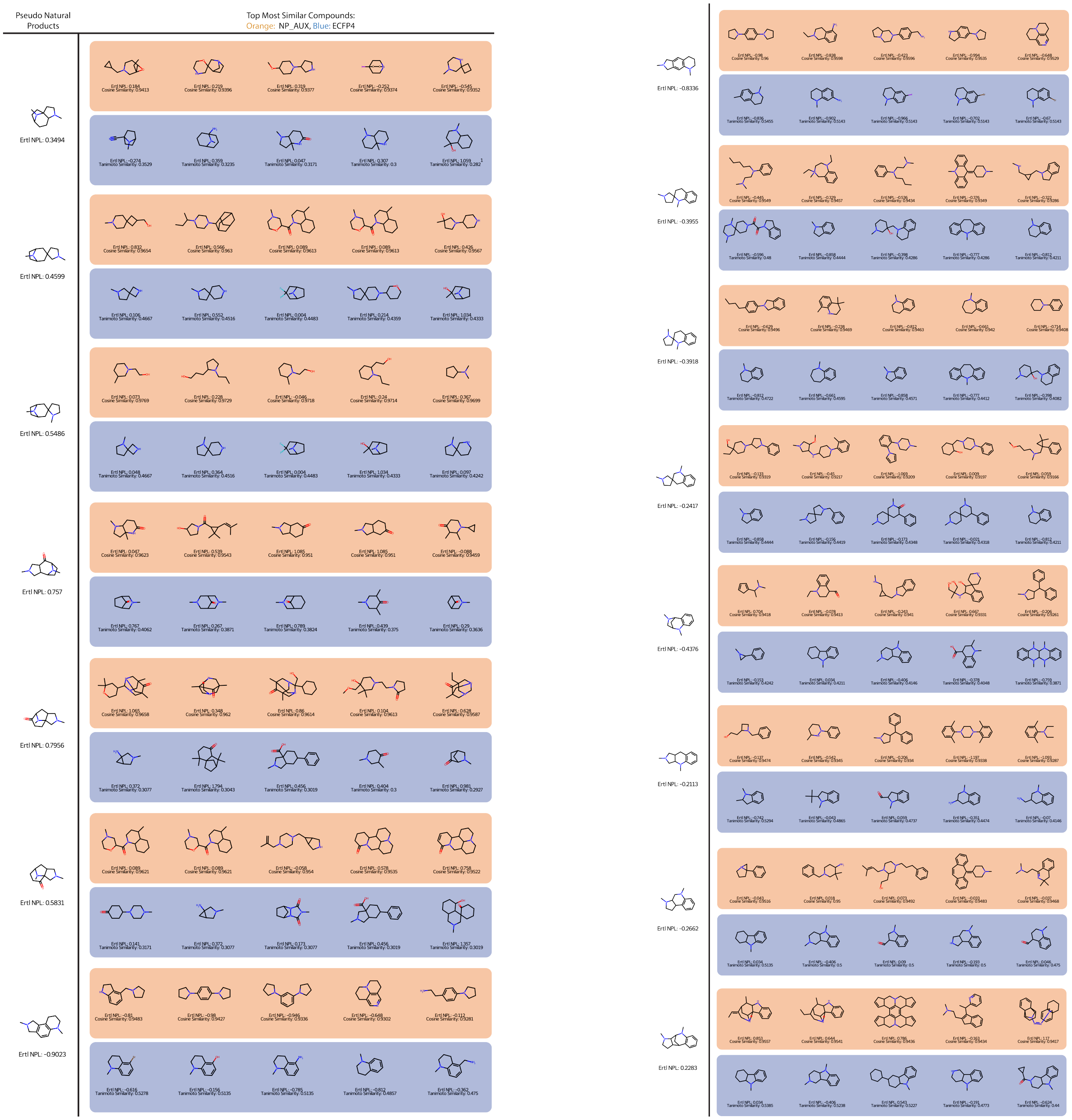


**Figure S1** Results of Similarity Search with 15 pseudo-natural products. For each pseudo-NP a similarity search was performed on the Zinc. The five most similar compounds are reported for the ECFP and neural fingerprint.
